# The Neuropilin-1/PKC axis promotes neuroendocrine differentiation and drug resistance of prostate cancer

**DOI:** 10.1038/s41416-022-02114-9

**Published:** 2022-12-22

**Authors:** Charly Blanc, Anissa Moktefi, Ariane Jolly, Pierre de la Grange, Denise Gay, Nathalie Nicolaiew, Fannie Semprez, Pascale Maillé, Pascale Soyeux, Virginie Firlej, Francis Vacherot, Damien Destouches, Mohamed Amiche, Stéphane Terry, Alexandre de la Taille, Arturo Londoño-Vallejo, Yves Allory, Jean Delbé, Yamina Hamma-Kourbali

**Affiliations:** 1 Univ Paris Est Creteil, INSERM, IMRB, 94010 Creteil, France; 2 AP-HP, Hôpital H. Mondor, Department of Pathology, 94010 Creteil, France; 3 Genosplice®, IM, Hôpital Pitié-Salpêtrière, Paris, France; 4 DLGBiologics, 75017 Paris, France; 5 SPPIN—Saints-Pères Paris Institute for the Neurosciences, Université de Paris, CNRS, 75006 Paris, France; 6 Univ Paris Est Creteil, UR TRePCa, 94010 Creteil, France; 7 AP-HP, Hôpital H. Mondor, Plateforme de Ressources Biologiques, 94010 Creteil, France; 8 Sorbonne University—CNRS, Institut de Biologie Paris-Seine, Laboratoire de Biogenèse des Signaux Peptidiques (BioSiPe), F-75252 Paris, France; 9 Faculty of Medicine, University Paris-Saclay, Le Kremlin-Bicêtre, France; 10 Research Department, Inovarion, Paris, France; 11 AP-HP, Hôpital Mondor, Department of Urology, 94010 Créteil, France; 12 Institut Curie, PSL Research University, CNRS UMR 3244, 75005 Paris, France; 13 Department of Pathology, Institut Curie, 92210 Saint-Cloud, France; 14 Institut Curie, PSL Research University, CNRS UMR 144, 75005 Paris, France

**Keywords:** Cancer, Prostate cancer

## Abstract

**Background:**

Neuroendocrine prostate cancer (NEPC) is a multi-resistant variant of prostate cancer (PCa) that has become a major challenge in clinics. Understanding the neuroendocrine differentiation (NED) process at the molecular level is therefore critical to define therapeutic strategies that can prevent multi-drug resistance.

**Methods:**

Using RNA expression profiling and immunohistochemistry, we have identified and characterised a gene expression signature associated with the emergence of NED in a large PCa cohort, including 169 hormone-naïve PCa (HNPC) and 48 castration-resistance PCa (CRPC) patients. In vitro and preclinical in vivo NED models were used to explore the cellular mechanism and to characterise the effects of castration on PCa progression.

**Results:**

We show for the first time that Neuropilin-1 (NRP1) is a key component of NED in PCa cells. NRP1 is upregulated in response to androgen deprivation therapies (ADT) and elicits cell survival through induction of the PKC pathway. Downmodulation of either NRP1 protein expression or PKC activation suppresses NED, prevents tumour evolution toward castration resistance and increases the efficacy of docetaxel-based chemotherapy in preclinical models in vivo.

**Conclusions:**

This study reveals the NRP1/PKC axis as a promising therapeutic target for the prevention of neuroendocrine castration-resistant variants of PCa and indicates NRP1 as an early transitional biomarker.

## Background

The mortality associated with prostate cancer (PCa) is mainly due to its progression to therapeutic-resistant metastatic disease. Androgen deprivation therapies (ADT) have improved the management of the disease; however, the vast majority of tumours ultimately acquire resistance to ADT. In most cases, this is associated with genomic alterations affecting the androgen receptor (AR) axis and apoptotic pathways [1]. Thus, reactivation of AR signalling contributes to tumour cell survival, proliferation, metastatic spread and to the development of castration-resistant prostate cancer (CRPC). However, other mechanisms have also been implicated in the development of CRPC as illustrated by the emergence of “non-AR-driven” neuroendocrine prostate adenocarcinoma (NEPC) [2].

Over a decade, a number of approved and promising therapies for CRPC have emerged, including taxane chemotherapies and AR pathway inhibitor strategies such as enzalutamide [3–5] and apalutamide, a next-generation AR inhibitor [6]. Compelling evidence suggests that prolonged treatment induces lineage crisis, associated with the progression of drug-resistant CRPC leading to PCa-related death [7]. Tumour cell acquisition of a neuroendocrine phenotype (NE) has been linked to drug resistance. Typically, NE differentiation (NED) is distinguished by reduced AR expression or activity as well as low expression of androgen-regulated genes (including PSA) and upregulation of NE markers [8, 9]. Recent studies have demonstrated that NEPC can be associated with recurrent genetic lesions, including loss of tumour suppressors, such as *RB1* [10] and p53 [10, 11], overexpression and genomic amplification of *MYCN* and *AURKA* [12, 13], and deregulation of epigenetic regulators/mediators such as BRN2 [14], REST [15] and EZH2 [12, 13], suggesting a late stage involvement. However, the work of others has shown that at least several of these key genes are upregulated early and may have roles in tumour cell transition to drug resistance [16].

We hypothesised that by examining the expression of a neuronal genes panel (Neurogenesis GO:0022008), we could identify drivers and potential therapeutic targets of NED and drug resistance. In comparing expression profiles of PCa tumours in cohorts comprising HNPC (54 patients) and CRPC (13 patients) phenotypes, we identified 92 neurogenesis genes within the CRPC cohort, several of which correlate with the NE PCa phenotype. Characterisation of this gene set identified the transmembrane glycoprotein Neuropilin-1 (NRP1).

NRP1 has been identified as an androgen-repressed gene whose expression is upregulated during the adaptive response to ADT [16]. Functional studies by this group revealed that NRP1 is likely involved in PCa metastatic migration via the upregulation of EMT genes. However, unrelated work has demonstrated that NRP1 has additional functions in development and potentially tumorigenesis [17, 18], some of which require PKC and AKT signalling upregulation [19].

In this report, we confirm that NRP1 is upregulated in transition to ADT, and further, our examination of human PCa datasets suggests that it may be present in 28% NEPC tumours. Importantly, we show that NRP1 is requisite for ADT transition in in vitro studies. Mechanistically, NRP1 induces expression and activation of the PKC pathway leading ultimately to increased cell survival. Finally, specific inhibition of the PKC pathway sensitises PCa cells to chemo-hormonal treatment. Together, our findings provide crucial insights into a novel NRP1/PKC axis to reveal promising new therapeutic targets in the treatment of PCa patients with NED and point to NRP1 as an early biomarker in tumour cell transition to the drug-resistant NE phenotype.

## Materials and methods

The source and catalog number of primary antibodies (Ab) are listed in Table S1. The experimental methods not described herein are provided in Supplementary Data.

### Human prostate cancer specimens

Prostate tissue samples were collected as part of an Institutional Review Board approved protocol at Henri Mondor Hospital in France. This study included 169 PCa patient samples (from radical prostatectomy) without having received prior hormone treatment at the institution (HNPC) and 48 CRPC tumours (collected by transurethral resection). CRPC tumours were separated in 27 CRPC-Adeno with less than 20% of neuroendocrine differentiation and 21 CRPC-NE with more than 20% of neuroendocrine differentiation as described [2, 8]. Immunohistochemistry of synaptophysin and chromogranin-A as NE markers were performed to attribute the percentage of NED. The study also included a few specimens derived from normal prostates from peritumoral tissues. Demographic, clinical and pathological parameters were collected prospectively in a database and retrospectively reviewed. Tumours were classified by the following criteria based on histomorphology by the genitourinary pathologist (Y. Allory).

### RNA microarray and transcriptomic data

Total RNA was isolated from frozen tissues using the miRNeasy kit (Qiagen) and transcriptome profiles were generated from HNPC (*n* = 54) and CRPC (*n* = 13) prostate cancer tissues and analysed using GeneChip® Human Transcriptome array 2.0 (ThermoFisher Scientific). Data have been analysed by Genosplice company (P. de la G range and A. Jolly) as previously described [20, 21].

### Immunohistochemistry

Immunohistochemistry (IHC) studies were performed as previously described [22] on formalin-fixed paraffin-embedded (FFPE) of all tissue samples. All slides were read by a genitourinary pathologist (A. Moktefi). For NRP1 staining analysis, the numerical score was assigned as no staining (0), low staining [1], moderate staining [2] and strong staining [3]. Staining was considered positive when the numerical score was ≥2 because normal glands are weak or negative.

### Cell culture

PCa cell lines (VCaP, LNCaP, 22Rv1, PC3 and DU145) were obtained from American Type Culture Collection and grown in RPMI-1640 (22Rv1, PC3, DU145, C4-2), DMEM (VCaP), or DMEM/RPMI (LNCaP) supplemented with 10% foetal bovine serum (FBS) (ThermoFischer Scientific, France). LNCaP-NE cells were obtained from LNCaP cells cultured in androgen-reduced conditions (phenol red-free DMEM/RPMI supplemented with 10% charcoal-stripped serum (CSS)).

Overexpression of NRP1 was obtained by stably transfecting LNCaP, C4-2 and 22Rv1 cell lines with the pCherry-mNrp1 plasmid (Addgene plasmid # 21934; [23] using lipofectamine 2000 (Invitrogen) standard protocol. Cells selection was performed in a medium containing G418 (300 µg/ml for C4-2 cells and 400 µg/ml for LNCaP and 22Rv1).

### Subcellular fractionation, western blot and immunoprecipitation

Cells were washed with cold PBS and lysed in 50 mM Tris buffer (pH 7.4) containing 150 mM NaCl, 1% Triton X-100, 1 mM EDTA and protease and phosphatase inhibitors cocktail (Roche). For cytosol and membrane proteins extractions, cells were prepared using a subcellular fractionation kit (Thermo Scientific) with protease and phosphatase inhibitors according to the manufacturer’s instructions.

For immunoprecipitation, proteins were prepared using lysis buffer (10 mM Tris pH 7.4, 150 mM NaCl, 1% Triton X-100, 5 mM EDTA, 10% glycerol, and protease and phosphatase inhibitors. Five hundred micrograms of total protein extract was incubated with 1 µg of anti-NRP1 antibody or control IgG overnight at 4 °C. Complexes were pulled down using Bio-Adembeads Protein A/G magnetic beads (Ademtech), washed with lysis buffer and analysed by SDS-PAGE. Immunostaining was visualised using the GBox system (Syngene). Band intensities were quantified using the Multi Gauge v3.0 software (Fujifilm).

### Small interference RNA assay

siRNA transfections for LNCaP, C4-2 and PC3 cells were performed using Lipofectamine RNaiMax (Invitrogen) according to the manufacturer’s protocol. Experimental conditions were optimised for LNCaP-NE. Briefly, LNCaP-NE cells were seeded in Poly-L-Lysine (Sigma) coated 12-wells at 70% confluence and then transfected with 200 pmol of either non-targeting siRNA 5’-GGUGCGCUCCUGGACGUAGCC-3’ as a control or target-specific NRP1 5’-GGCUACGUCCAGGAGCGCACC-3’ or PKC isoforms siRNAs with Lipofectamine Messenger MAX (Invitrogen). PKCα, PKCε and PKCδ—siRNA were a gift from Dr K. Mahéo (Inserm UMR 1069, Tours, France) and are referenced [24]. Transfection efficiency was evaluated by western blot analysis.

### Xenograft studies

All mouse experiments were performed according to guidelines on animal care and with appropriate institutional certification of ethical comity and conducted in compliance with European Community. LNCaP (2 × 10^6^ in 50% Matrigel) or PC3 (2 × 10^6^) cells were injected subcutaneously into the right flank of 5-week-old male NMRI nude mice (Janvier, Le Genest-Saint-Isle, France). In the Enzastaurin/castration combination experiment, mice bearing LNCaP tumours of about 200–300 mm^3^ were separated randomly into several groups. Mice were then castrated by bilateral orchiectomy and treated one day after castration with 100 mg/kg Enzastaurin or vehicle by oral gavage every day. In the Enzastaurin/docetaxel combination experiment, mice bearing PC3 tumours of about 50–80 mm^3^ were separated randomly into several groups and treated with 100 mg/kg Enzastaurin or vehicle by oral gavage every day and/or with 5 mg/kg docetaxel at or PBS vehicle by intraperitoneal injection once a week. Tumour size was measured two times per week with a caliper and tumour volume was calculated with the formula: V = 4/3π*R1^2^*R2 whereby radius 1 (R1), radius 2 (R2). Then, the percentage of tumour size was assigned to 100% at the beginning and each measure represents the percentage of tumour growth evolution.

### Analyses of single-cell RNAseq datasets

ScRNAseq analysis of primary prostate tumours [25] was undertaken using the publicly available web tool at www.pradcellatlas.com. The epithelial atlas was explored in this work.

To analyse and compare results with publicly available scRNAseq data from a castration-resistant LNCaP cell model [26], datasets from GSE205765 were downloaded from GEO, transformed into Seurat objects, MT levels set at 10% for each object and objects integrated using Seurat pipelines [26]. Clusters exhibiting high mitochondrial or ribosomal signatures were considered non-viable and removed from the analysis. The Find Clusters resolution parameter was set low to generate clusters with only fundamental transcriptional differences. Cluster (c)0 included both FCS and CSS cells (33%, 52%, respectively), c1 was almost exclusively FCS (60%, 1%, respectively), c2 almost exclusively CSS (7%, 47%) as previously shown. Dotplot analysis uses a Seurat tool.

### Statistical analysis

Pearson correlations were implemented for gene-gene expression correlation using GraphPad Prism (GraphPad Software). In bar graphs and dose-response curves, comparisons between each group were performed using Student’s *t*-test or multiple *t*-test. All statistical tests used a two-tailed α = 0.05 level of significance and were performed using GraphPad Prism (GraphPad Software). For in vitro studies, graphs show pooled data with error bars representing ± SEM obtained from at least three independent experiments. Statistical significance was accepted for *P < 0.05; **P < 0.01; ***P < 0.001; ****P < 0.0001.

## Results

### Validation of the Mondor Patient dataset and identification of a neuronal transcriptional programme within the CRPC subset

**Fig. 1:**
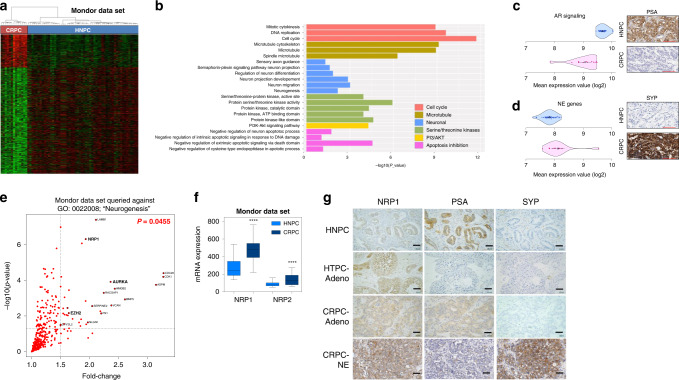
The NE profile is increased in CRPC compared to HNPC. **a** Heatmap showing differential regulation of 1849 genes across 13 CRPC and 54 HNPC patients (Fold change ≥ 1.5; *p*-value ≤ 0.05). See also Supplementary Tables S2 and S3. **b** Functional bar graph from DAVID gene ontology analysis (https://david.ncifcrf.gov) of Mondor dataset CRPC upregulated genes. *P*-values are represented as bars. **c**, **d** Left panels show violin plots of abundance of mRNA transcripts in **c** AR signalling [29], or **d** NEPC signature [2]. Dots represent patients; diamonds and solid lines represent mean and 95% confidence interval, respectively. See also Supplementary Tables S4 and S5. Right panels show immunohistology staining for **c** PSA or **d** SYP protein expression in HNPC and CRPC samples respectively. Scale bars, 100 µm. **e** Scatter plot shows significantly upregulated genes associated with “Neurogenesis” pathway (GO:0022008) in Mondor dataset CRPC compared to HNPC samples. See also Supplementary Table S6. **f** Boxplot shows gene expression of *NRP1* in 54 HNPC and 13 CRPC tumours (Mondor Dataset), as measured by transcriptomic array. **g** Representative IHC for NRP1, PSA and SYP in HNPC and different CRPC tumours. Scale bars, 50 µm. See also Fig. S1.

A neuroendocrine (NE) phenotype correlates with aggressive treatment-resistant tumours in treatment-resistant prostate cancer. Rigorous sets of signature genes have been identified, including CHGA, SYP, TUBB3, EZH2 ([27, 28]). To identify potential novel candidates involved in the transition from a CRPC-adenocarcinoma to NE phenotype in ADT-resistant patients’ tumours, whole-transcriptome profiles were analysed using oligonucleotide microarrays from 13 CRPC and 54 localised HNPC tumours (Mondor Dataset). Clinical characteristics are summarised in Table S2. Comparative analysis between CRPC and HNPC showed a significant differential expression in 1849 genes (Heatmap, Fig. 1a and Table S3). Examination of CRPC upregulated genes, using the functional annotation tool DAVID, showed that many were involved in cell cycle, microtubule alterations, negative regulation of apoptosis as expected, as well as serine/threonine protein kinase and neuronal signatures (Fig. 1b).

Examination of the Mondor dataset for AR-regulated genes using a signature previously defined by Hieronymus et al., 2006 [29] (see Table S4) revealed an overall lower expression in CRPC as compared to HNPC (*p* = 0.00042; Fig. 1c, left). This result was confirmed by immunohistochemistry (IHC) with lower protein expression of androgen-responsive gene PSA in CRPC compared to HNPC tissues (two representative samples, Fig. 1c, right, and data not shown). Concomitant with the decrease in AR-targeted genes, we observed a modest increase in the CRPC-NE profile using an NEPC signature ([2], see Table S5) (Fig. 1d).

We hypothesised that some overlapping members between a defined neurogenesis signature (GO:0022008) and the Mondor dataset might provide candidates for PCa transition to an NE phenotype. Examination of the Mondor dataset against this signature revealed upregulated expression of *NRP1*, *NRP2, AURKA*, *EZH2, LAMB1*, *NLGN1* genes (Fig. 1e, Table S6), several of which have already been identified in the NE phenotype (*AURKA, EZH2, NLGN1)*.

### NRP1 is an early induction gene for NED

We decided to focus on NRP1 in NED because it has been linked to high Gleason score and ADT [16] and functionally linked to the regulation of PKC and AKT pathways [19, 30, 31], both observed in the DAVID analysis (Fig. 1b).

To validate it as a potential candidate, we observed that NRP1 expression was upregulated in the CRPC Mondor dataset (Fig. 1f, Fig. S1A), downregulated against AR genes within the Mondor dataset (Fig. S1B) and showed increased protein expression from HNPC to CRPC-NE stages (Fig. 1g, Fig. S1C).

To further examine the timing and location of NRP1 in PCa, we relied upon a publicly available single-cell RNAseq (scRNAseq) analysis of 13 tumour biopsies from 12 PCa patients, including those with luminal, basal, or proliferative phenotypes [25] for details). Figure S2A shows an integrated UMAP of clusters based on gene similarity: cluster 10 represents basal, cluster 12 proliferative and all other clusters luminal epithelial phenotypes. The authors categorised basal and luminal clusters as mainly non-malignant/unresolved and malignant/unresolved, respectively. Using their online interactive tool to investigate genes of interest (www.pradcellatlas.com, Epithelial dataset), we found that NRP1 and other neuronal genes from the neurogenesis signature comparison (Fig. 1e) were similarly expressed across luminal clusters in most patients and absent from either basal or proliferative clusters. Interestingly, most were absent from clusters 2 and 7, derived primarily from a single patient, which express high levels of *AR* and *KLK3*. These results confirm that NRP1 is expressed even before post-operative treatment.

To ask about NRP1 expression after ADT, we examined the SU2C-PCF dataset (208 CRPC mRNA samples across both Adeno-CRPC and NEPC (11%), [32]. Clusterplots show NRP1 expression was upregulated in ~12% of samples (Fig. S2C). Co-expression of NRP1 and different NE signature genes revealed that although overall co-expression against CHGA, SYP, TUB33, EZH2, TP53 showed low to negative Spearman scores, other NE genes correlated positively across the entire cohort and most co-expression analyses revealed at least some samples with positive correlation (Fig. S2C).

To better focus on NE samples only, a cohort of 39 patients (37 mRNA samples) with high NEPC and low AR scores (see Table S7) was selected from the SU2C-PCF dataset. Of the 37 samples, 8 expressed NRP1 mRNA (Fig. S3).

To try to define a true NEPC population, we examined the expression of CHGA, SYP and EZH2, all NE signature genes, and found 14 samples positive for all 3 genes. Of these 14, 4 samples also expressed NRP1 (Fig. S3). These combined results confirm upregulated expression of NRP1 in 12% CRPC samples overall, increased (22%) within a selected NEPC-High cohort. Further, within our putative NEPC population, ~29% co-expressed NRP1.

We then examined NRP1 expression in the LNCaP NED model, in which LNCaP-NE cells emerge from a long-term culture of LNCaP cells in an androgen-deprived medium [33, 34].

**Fig. 2:**
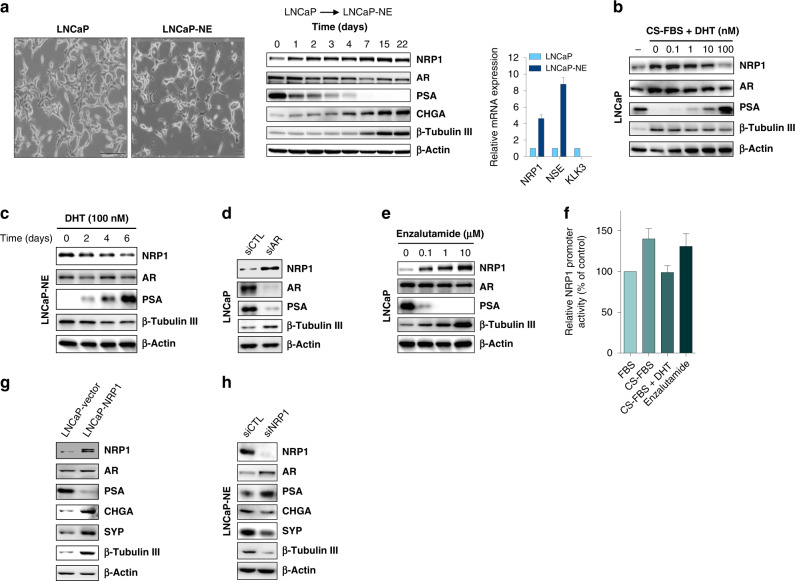
NRP1 promotes NED through regulation of the AR axis. **a** Left. Photos of LNCaP cutures, control (Left), after androgen deprivation (Right). Scale bars, 200 µm. Middle. Western blot analysis of NRP1, AR, PSA and NE markers CHGA and β-Tubulin III in LNCaP cells over time after androgen depletion. Right. qPCR of *NRP1*, *KLK3* and NE marker *NSE* in control (light blue) and androgen-deprived LNCaP (dark blue) at Day 4 after androgen deprivation. **b** Western blot of NRP1, AR, PSA and NE marker β-Tubulin III from LNCaP cells after androgen depletion (CS-FBS) followed by DHT treatment at indicated doses and times (96 h). **c** Western blot of NRP1, AR, PSA, β-Tubulin III from LNCaP-NE cells during DHT treatment over time (see indicated doses and times). **d** Western blot of NRP1 and other proteins in LNCaP cells after siRNA knockdown of AR. Non-targeting siRNA is siCTL. **e** Western blot of NRP1 and other proteins after treatment of LNCaP cells with stated concentrations of enzalutamide. **f** NRP1 promoter activity in LNCaP cells after AR pathway inhibition (CS-FBS and enzalutamide) or activation with DHT as measured by luciferase assay. **g** Western blot for NE markers and AR axis proteins from LNCaP cells stably overexpressing NRP1-containing vector compared with empty vector (LNCaP-vector). **h** Western blot of NE markers in LNCaP-NE cells following siRNA knockdown of NRP1.

As expected, NRP1 protein levels were low in epithelial cell lines LNCaP, VCaP C4-2, 22RV1 and DU145 and moderate to high in cell lines displaying pronounced NE phenotypes such as LNCaP-NE, C4-2-NE, or small cell NE-like PC3 cells (Fig. S4A). LNCaP-NE cells were validated by morphological changes, downregulation of androgen-regulated genes (*KLK3*/PSA) and upregulation of NE markers such as CHGA, neuron-specific Enolase (NSE) and β-Tubulin III (Fig. 2a). Interestingly, basal NRP1 protein levels rose very early in the time course and remained elevated over time (Fig. 2a).

As previously observed [16], we found that NRP1 expression is negatively regulated by the AR pathway. Treatment of LNCaP-NE and C4-2-NE cells with dihydrotestosterone (DHT) strongly reduced the expression of both NRP1 and NE markers and increased the androgen-regulated protein PSA (Fig. 2b, c and Fig. S4B). Inversely, the knockdown of AR in LNCaP or C4-2 cells increased NRP1 expression, as well as NE marker β-Tubulin III, compared to the non-targeting siRNA control (Fig. 2d and Fig. S4C). Similar results were observed upon treatment of cells with the AR inhibitor enzalutamide (Fig. 2e and Fig. S4D). Importantly, AR impacts NRP1 promoter activity since this activity was increased by enzalutamide or androgen-depleted condition and decreased by treatment with DHT (Fig. 2f).

To ask if NRP1 expression might be required for the NE phenotype, we stably transfected LNCaP cells with an NRP1-expressing vector. Western blot analysis revealed that these cells displayed a neuronal morphology with upregulation of NE markers, and downregulation of androgen-regulated protein PSA (Fig. 2g). Conversely, knockdown of NRP1 expression using siRNAs diminished NE marker expression in LNCaP-NE cells (Fig. 2h). Exogenous expression of NRP1 in 22Rv1 and C4-2 cells, further confirmed this link (Fig. S4E and F). These results demonstrate for the first time that NRP1 expression is directly associated with the NED process and may be an early requisite for transition.

### NRP1 drives NED through the PKC signalling pathway

**Fig. 3:**
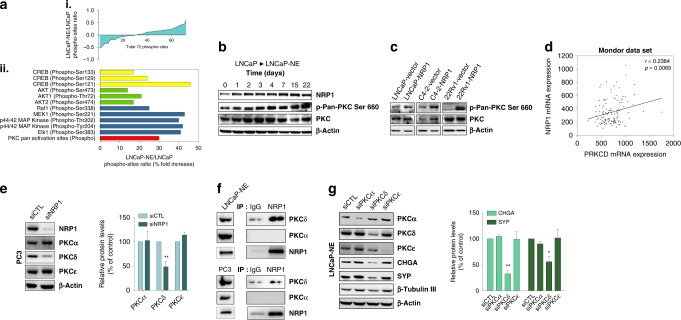
NRP1 promotes NED through the PKC pathway. **a** (i) Phosphorylation status of all screened proteins based on a phospho-specific protein microarray analysis. **a** (ii) Phospho-specific protein microarray data shows fold change of indicated phosphoproteins in NED after normalisation to total protein expression. **b** Western blot of LNCaP during NED (upon androgen depletion) shows phosphorylation of Pan-PKC (S660). **c** Western blot shows Pan-PKC (S660) phosphorylation from LNCaP, C4-2 and 22Rv1 cells stably transfected with NRP1 vs empty vector. **d** Scatter plots show correlation between *NRP1* and *PRKCD* mRNA from Mondor clinical cohort. **e** Western blot of PKC isoforms from PC3 cells transfected with siNRP1 or siCTL. Bar graph (Right) shows relative protein levels as % control. **f** Western blots of anti-NRP1 or control IgG LNCaP-NE Immunoprecipitates blotted for PKCα, PKCδ or NRP1 (Left panels). Total lysate blotted for PKCα, PKCδ or NRP1 shown in the right panel. Data are represented as mean ± SEM; *p*-value by two-tailed unpaired *t*-test. *P < 0.05; **P < 0.01; ***P < 0.001. **g** Western blot of PKC isoforms and NE markers in LNCaP-NE cells transfected with non-targeting siRNA (siCTL) or PKCα, PKCδ, PKCε siRNA. Bar graph (Right) shows relative protein levels of CHGA or SYP as % control.

NED-inducing stimuli have been shown to increase intracellular levels of cAMP for activation of the transcription factor cAMP response element-binding protein (CREB) [35, 36]. We explored phosphorylation pattern differences in LNCaP and LNCaP-NE cells using a CREB pathway phospho-antibody array containing 174 antibodies. Overall, more proteins were found to be phosphorylated in LNCaP-NE than in LNCaP cells (Fig. 3a) and several key components of CREB, AKT and ERK pathways were phosphorylated in NED (Fig. 3a), as previously reported [37].

The phospho-specific protein microarray analysis also indicated increased phosphorylation within the PKC pathway in LNCaP-NE cells compared to LNCaP cells (Fig. 3a). We examined this pathway in greater detail because NRP1 function has been linked to PKC activation [38]. DAVID analyses confirmed the importance of Ser/Thr pathways and *PRKCD* was identified in the Mondor CRPC upregulated genes list (Fig. 1b, Table S3). We confirmed increased expression of PKC and increased phosphorylation at PKC pan-activation site Ser660 by Western blot analysis following a time course of androgen depletion in LNCaP cells (Fig. 3b). NRP1 overexpression in LNCaP, C4-2 or 22Rv1 cells also resulted in increased PKC phosphorylation compared to control vector-transfected cells (Fig. 3c), suggesting that PKC activation might be NRP1-dependent. In support of this, a variety of downstream PKC targets were examined during the transition from LNCaP to LNCaP-NE phenotype and in stably transfected 22Rv1 cells overexpressing NRP1 (Fig. S5). Both ERK and Akt exhibited increased phosphorylation and notably, apoptosis inhibitor Bcl-2 was upregulated (Fig. S5).

PKCs form a large family, including the widely characterised isoenzymes (PKCα, PKCβ, PKCγ, PKCδ, PKCε) expressed in multiple cancers and during neuronal differentiation [39]. Examination of the Mondor dataset showed that transcription of *PRKCA* and *PRKCD* were significantly upregulated in CRPC compared to HNPC tumours (Fig. S6A). Examination of a scRNAseq dataset comparing hormone-intact (FCS) and castrate-condition (CSS) LNCaP cells [26], GSE205765, Fig. S6B) confirmed upregulation of *PRKCA* and *PRKCD* in CSS clusters only (See Materials and Methods for details). Upregulated phosphorylation was also observed in PKCα, PKCδ and PKCε proteins from LNCaP-NE compared to LNCaP cells (Fig. S6C). Finally, because PKC is activated at the plasma membrane, we examined the distribution of NRP1 and isoforms PKCδ and PKCα in cytosol and membrane fractions of LNCaP and LNCaP-NE cells. High levels of EGFR or GAPDH in the membrane or cytosol fractions, respectively, showed fraction purity (Fig. S6D left). As expected, NRP1 was upregulated in LNCaP-NE cells and primarily localised to the cell membrane. Expression of both PKCδ and PKCα isoforms was also upregulated in LNCaP-NE cells and primarily localised to the cell membrane (Fig. S6D right).

We next examined the relationship between NRP1 and PKC isotypes in NED. Cross-correlation of NRP1 and PKCD in the Mondor dataset suggested that increased transcription was tightly coupled (Fig. 3d). Further, NRP1 transcriptional silencing in PC3 cells, which strongly expressed this protein, resulted in a significant reduction in PKCδ protein levels but did not change PKCα and PKCε levels (Fig. 3e). Finally, co-immunoprecipitation experiments revealed that NRP1 capture resulted in co-IP of PKCδ in LNCaP-NE and PC3 cells (Fig. 3f). Although NRP1 activation of the PKC pathway in endothelial cells requires VEGF and VEGFR co-receptors [38], examination of the GSE205765 scRNAseq dataset found none of these mediators in either FCS or CSS condition (data not shown). Taken together, these results indicate that NRP1 positively regulates PKC expression and activation in NED and may directly associate with specific isoforms in the cell membrane.

To ask which PKC isoform(s) might be requisite for the NE phenotype, siRNAs were used to target PKCα, PKCδ or PKCε in LNCaP-NE cells, and NE profile markers examined by western blot. Knockdown of PKCδ resulted in a significant reduction of CHGA and SYP, suggesting a reversal of the NE phenotype (Fig. 3g).

We also observed co-expression of PKCα and PKCδ and downstream target BCL2 in our SU2C/PFC NEPC NRP1 + population (Fig. S3), confirming the existence of this phenotype in human advanced PCa samples.

Together, these results suggest that PKC expression is linked to NRP1 and that PKC might be a significant contributor to the NE phenotype.

### Inhibition of the PKC pathway counteracts NED and blocks CRPC progression in vivo

**Fig. 4:**
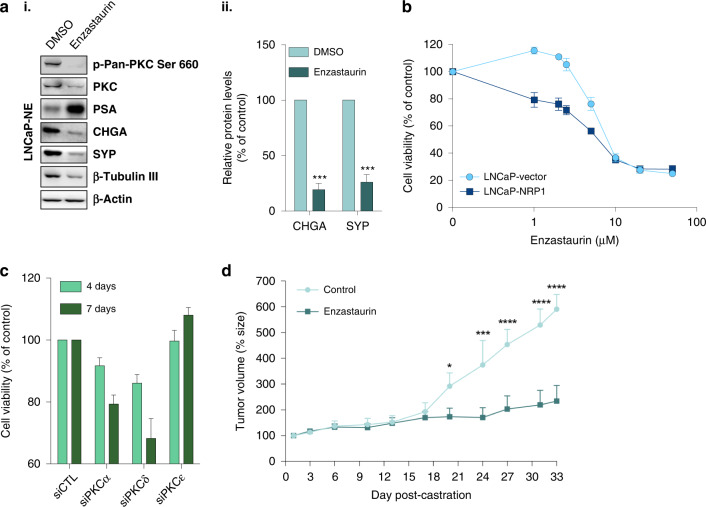
PKC promotes cell survival in NED. **a** (i) Western blot for NE markers in LNCaP-NE after treatment with DMSO (Left), enzastaurin (Right) for 4 days at 5uM. **a** (ii) Bar graph shows relative protein levels of CHGA, SYP in DMSO- or Enzastaurin-treated LNCaP-NE as % of control. **b** Line graph shows timeline of cell viability of stably transfected LNCaP cells overexpressing NRP1 (squares) or with empty vector (circles) after enzastaurin treatment. **c** Bar graph shows viability of LNCaP-NE cells after transfection with siRNA targeting PKCα, PKCδ and PKCε or non-targeting siRNA (siCTL). **d** Line graph shows LNCaP tumour size over time in nude mice after LNCaP ectopic xenografting, castration and daily treatment with Enzastaurin (squares) or no treatment (circles). *N* = 7 for all conditions. See Materials and Methods for details. Data are represented as mean ± SEM; *p*-value by two-tailed unpaired *t*-test. *P < 0.05; **P < 0.01; ***P < 0.001; ****P < 0.0001.

Based on the findings that PKC expression and activation are upregulated early in NED and could drive NE transdifferentiation (Fig. 3), we postulated that treatment with the PKC inhibitor Enzastaurin would reverse NED. Although initially described as a PKCβ inhibitor, enzastaurin has a broad impact on other PKC isoforms, including PKCα and PKCδ, and is frequently used as a pan-PKC inhibitor [40–42]. Treatment of LNCaP-NE cells with Enzastaurin resulted in decreased phosphorylation of pan-PKC and significantly reduced expression of NE markers (Fig. 4a). LNCaP cells stably overexpressing NRP1 (LNCaP-NRP1) exhibited reduced viability in the presence of Enzastaurin compared to control LNCaP cells with a decreasing GI50 from 8.9 to 5.3 µM (Fig. 4b). SiRNA knockdown of PKC isoforms in LNCaP-NE cells confirmed their diminished viability of through PKC activity (Fig. 4c).

We then evaluated the combined effect of castration and Enzastaurin on tumour growth in vivo using LNCaP ectopic xenograft model. The LNCaP model is commonly used in vitro and in vivo to model the response to ADT of PCa. Xenografted male mice were castrated by surgery to block androgen synthesis and promote an apparent LNCaP-NE phenotype as defined by drug resistance in injected LNCaP tumours. In response to castration, tumour growth in control mice remained low for two weeks, after which time tumour cells proliferated despite castration, and developed androgen-independent tumours (Fig. 4d). Enzastaurin treatment resulted in significant inhibition of tumour growth over 30 days post-castration (Fig. 4d). Taken together, these and above results support the implication of a NRP1/PKC axis in CRPC that promotes the survival of NE cells. They further indicate that Enzastaurin treatment can counter PCa progression.

### The NRP1/PKC axis confers resistance to taxane chemotherapy

**Fig. 5:**
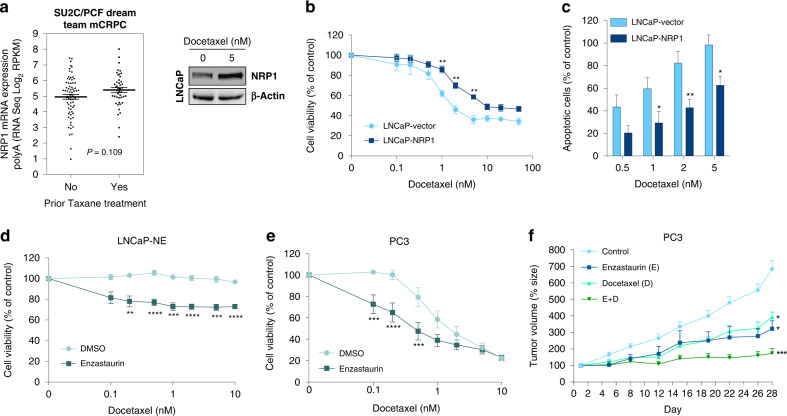
NRP1 overexpression leads to docetaxel resistance that is reversed through PKC inhibition. **a** Left: Dotplot comparisons of *NRP1* mRNA expression in mCRPC samples (*n* = 118) prior to (Left) or after (Right) Taxane treatment. Data taken from Stand Up To Cancer (SU2C)/Prostate Cancer Foundation (PCF) Dream Team dataset [43]. Data analysed using cBioPortal. Right: Western blot of NRP1 in LNCaP cells 72 h after docetaxel treatment. **b** Dose-response curves show viability of stably transfected LNCaP clones overexpressing NRP1 or control vector after 72 h incubation with docetaxel (doses indicated on *X*-axis). **c** Bar graph shows % apoptotic LNCaP cells stably overexpressing NRP1 (dark blue) or control vector (light blue) after 72 h incubation with different doses docetaxel (doses indicated on *X*-axis). **d**, **e** Dose-response curves of **d** LNCaP-NE or **e** PC3 cells after docetaxel treatment at indicated doses with (Squares) or without (Circles) 5 µM enzastaurin. **f** Time course of PC3 tumour volume increase from PC3 cells ectopically xenografted into nude mice treated daily with glucose 5% (Circles, *n* = 6), enzastaurin (Squares, *n* = 5), weekly with docetaxel (Blue Triangles, *n* = 6) or a combination of treatments (enzastaurin and docetaxel, Green Triangles, *n* = 8). See Materials and Methods for details.

We have previously reported that CRPC cells with upregulated NE phenotype are resistant to a wide range of cytotoxic agents, including taxane chemotherapies [34]. Moreover, analysis of RNA-sequencing data of 150 metastatic CRPC bone or soft tumour biopsies from the Robinson cohort [43] showed a trend toward increased expression of NRP1 in patients treated with taxane chemotherapies (Fig. 5a). In vitro, docetaxel treatment of LNCaP cells induced the expression of NRP1 in cells that survive following treatment (Fig. 5a). These results strongly suggest a role for NRP1-induced NED in resistance to docetaxel. To answer this question, LNCaP-NRP1 and LNCaP-vector cells were treated for 72 h with docetaxel and cell viability was measured by MTT. NRP1 overexpression resulted in a lower sensitivity to docetaxel with an increase of GI_50_ from 1 to 4 nM (Fig. 5b). Similar results were obtained by overexpressing NRP1 in 22Rv1 and C4-2 cell lines (Fig. S7A and S7B). In addition, NRP1 overexpression in LNCaP cells conferred resistance to docetaxel-induced apoptosis in a dose-dependent manner (Fig. 5c). Notably, NRP1 overexpression in LNCaP cells also increased the expression of Bcl-2, β-Tubulin III and MDR-1 in LNCaP-NRP1 compared with LNCaP-vector cells (Fig. S7C), all known to be implicated in cell survival and taxane resistance [44, 45].

The PKC pathway has also been shown to play a role in chemotherapeutic resistance [46]. Enzastaurin treatment significantly enhanced the anti-tumoral effect of docetaxel on LNCaP-NE and metastatic, castration-resistant PC3 cells (Fig. 5d and e). PC3 cells are documented to have an NE phenotype and have been used in this context by others [47, 48]. In in vivo studies, a combination of Enzastaurin with docetaxel resulted in a stronger inhibitory effect on xenografted PC3 tumour growth when compared with single-agent treatments (Fig. 5f). These results indicate that Enzastaurin treatment may counteract taxane resistance and potentially enhance the response to docetaxel in CRPC-NE.

## Discussion

Our results showed that NRP1, a transmembrane glycoprotein expressed in a wide variety of human cancers, and required for aggressive tumour growth and tumour-related angiogenesis (reviewed in ref. [49], is an important early player in PCa drug resistance and a putative candidate for its induction. Our analyses of expression profiles from human PCa specimens (Mondor dataset) identified NRP1 as a central feature amongst the CRPC cohort overexpressed NE-related genes. We found that NRP1 was also detectable in HTPC-adeno and CRPC-Adeno specimens (data not shown) and primary luminal tumour samples [25], indicating that low NRP1 expression precedes transition to drug resistance. In agreement with others [16], we also observed that androgens maintain NRP1 at low levels and that release from their effects promotes strong upregulation of both NRP1 transcription and protein expression. Importantly, our NRP1 overexpression and knockdown studies showed for the first time that NRP1 may be a key player in the transition to therapy resistance. Its upregulation during ADT transition and potential role(s) in the induction of ADT-NE indicate NRP1 as a novel biomarker and a target for more efficacious therapy to prevent PCa drug resistance.

Our examination of the human SU2C-PCF CRPC dataset revealed that NRP1 and downstream PKC players are expressed in a subset of NEPC tumours. Others have observed plasticity in advanced PCa associated with epigenetic reprogramming driven by N-Myc [50]. Thus, multiple NEPC subtypes likely exist reflecting this (and potentially other unrelated) epigenetic transitions. We postulate that, based upon early expression of NRP1 in NE transformation, it will play a seminal role in NE transformation upstream of further alterations.

Because of its limited molecular characterisation, there is no standard treatment for patients with NEPC. Although multiple pathways, including PI3K/AKT and MAPK, likely converge to drive the emergence of NE phenotype, no studies so far have demonstrated clear clinical benefits from targeting these pathways [51]. In our CRPC-NE models, inhibition of these pathways with specific inhibitors **(**PI3K inhibitor LY294002 and ERK inhibitor PD0325901) have failed to reverse NE phenotype (data not shown), suggesting that these pathways may not have a direct impact on NRP1-induced NED.

Our results show that NRP1 upregulation correlates with PKC activation as determined by increased phosphorylation during ADT and that NRP1 co-immunoprecipitates with PKC, suggesting a functional link. Further, both NRP1 overexpression and knockdown studies support the notion that NRP1 upregulation during ADT is directly required for PKC activation. Work in unrelated fields has shown that NRP1 directly associates with VEGFRs for VEGF-mediated PKC induction during angiogenesis [38] We have not identified either VEGFRs or VEGF in our cultures (data not shown), but NRP1 is a well-recognised pleiotropic receptor and has been shown to associate with plexins, PDGFRs, leptin, etc. to mediate numerous downstream functions [52, 53]. Identification of specific NRP1 co-receptors and ligands for PKC induction in drug-resistant PCa is a current focus of our lab.

Microarray data from the patient cohort (Mondor dataset) showed that PKCα and PKCδ were most highly expressed in human CRPC, and our in vitro results and examination of a scRNAseq database [26] confirmed this finding. While our in vitro data support a clearer role for PKCδ in NRP1-induced NED, PKCα may also play a role in cell survival, as demonstrated in knockdown studies. These results warrant further studies to clarify this point.

PKC pathway activation in response to androgen deprivation has been shown to promote resistance to AR-targeted therapy [54]. We found that PKCα or PKCδ knockdown significantly decreased LNCaP-NE survival in vitro and that in vivo treatment with enzastaurin, a powerful pan-PKC inhibitor, of castrated mice injected with LNCaP tumour cells resulted in a significant reduction of tumour growth. Both results support a role for PKC activity in tumour cell viability. Importantly, a comparison of control and NRP1-overexpressing LNCaP cells after enzastaurin treatment revealed that only NRP1-overexpressing cells were susceptible to treatment resulting in reduced viability, thus further confirming an important role for NRP1 in PKC activation.

For decades, taxane-based chemotherapies have been the main treatment for metastatic CRPC. Although it prolongs overall survival for some patients, many do not respond to treatment, while others invariably develop resistance. We have previously reported that CRPC-NE cells are resistant to multiple cytotoxic agents [34]. In our in vitro study, we demonstrated that NRP1 promotes higher resistance to docetaxel-based chemotherapy concomitantly with the acquisition of NE phenotype.

Numerous cellular pathways involving apoptosis, signalling components, drug efflux pumps and tubulin are implicated in the development of chemoresistance [55]. Both NRP1 and the PKC pathway have been implicated in drug resistance in multiple cancers [46]. Here, we have shown that NRP1 overexpression in LNCaP cells induces the expression of some key players in cell survival and taxane resistance, including Bcl-2, β-Tubulin III and MDR-1. All these are known PKC downstream targets [56]. We also show that Enzastaurin increases the cytotoxic effects of docetaxel in CRPC-NE cells in vitro and in a preclinical model in vivo. Altogether, these findings point to an important NRP1/PKC axis that promotes tumour cell survival and docetaxel resistance.

## Conclusions

While several aspects of therapy-resistant treatment-induced NEPC have been explored, how to therapeutically target these aggressive metastatic NE subsets remains a clinical challenge [51]. We propose that PKC inhibitors could be used as novel co-targeted therapies in an adjuvant setting combined with AR-directed therapy and cytotoxic chemotherapy in the treatment and/or prevention of multi-resistant CRPC-NE disease. The clinical potential of targeting the PKC pathway with Enzastaurin has been demonstrated in neuroendocrine pancreatic cancer [57]. In the prostate cancer setting, however, a phase II trial evaluating Enzastaurin in combination with docetaxel for patients with PSA progression in CRPC was disappointing so far that it showed no benefits in combination [58]. Nevertheless, it remains that PSA progression in castrate state may not be an ideal inclusion criterion because CRPC-NE most likely displays reduced if no expression of PSA. In such studies, it would be interesting to include patients with a high CRPC-NE contingent.

Our work reveals several novel findings with implications for patients with CRPC and drug-resistant NE disease. These findings support a real promising clinical value of the NRP1/PKC-targeted axis in the treatment and prevention of therapy-resistant treatment-induced NED. NRP1 would provide an excellent biomarker of PCa progression and particularly early diagnosis of NE disease.

## Supplementary information

Supplementary methods

aj-checklist

NC3Rs ARRIVE Guidelines Checklist

Supplementary Figure 1

Supplementary Figure 2

Supplementary Figure 3

Supplementary Figure 4

Supplementary Figure 5

Supplementary Figure 6

Supplementary Figure 7

Supplementary Table-S1

Supplementary Table-S2

Supplementary Table-S3

Supplementary Table-S4

Supplementary Table-S5

Supplementary Table-S6

Supplementary Table-S7

## Data Availability

Data generated and analysed during this study are included in this published article and its supplementary information files. Other datasets used during the current study are available from the corresponding author on reasonable request. HTA2.0 data have been deposited to the NCBI Gene Expression Omnibus (GSE200879).
