# Supplementary material for: The Neuropilin-1/PKC axis promotes neuroendocrine differentiation and drug resistance of prostate cancer
Source: Br J Cancer. 2022 Dec 22;128(5):918–27. doi: 10.1038/s41416-022-02114-9 (PMC9977768; doi:10.1038/s41416-022-02114-9)
Supplement: Supplementary file 1 — Supplementary methods [file 41416_2022_2114_MOESM1_ESM.docx]

**The Neuropilin-1/PKC Axis Promotes Neuroendocrine Differentiation and Drug Resistance of Prostate Cancer**

**SUPPLEMENTARY MATERIALS AND METHODS**

**Phosphoprotein Profiling with Phospho Explorer Antibody Microarray**

CREB Pathway Phospho Antibody Array was designed and manufactured by Full Moon Biosystems, Inc., and contains 174 antibodies. Each antibody had six replicates that were printed on a coated glass microscope slide together with multiple positive and negative controls. The antibody array experiment was performed according to the manufacturer’s established protocol and analyzed using Microarray scanner (Tecan).

**qRT-PCR**

Total RNA was extracted using RNeasy Mini Kit (Qiagen) according to the manufacturer’s instructions. For RT-PCR experiments, cDNA was retrotranscribed using High Capacity cDNA Reverse Transcription Kit (ThermoFischer Scientific) according to supplier’s instructions. qPCRs were performed using Maxima SYBR Green/ROX qPCR Master Mix (Thermo Scientific) on a StepOnePlus Applied Biosystems Real Time PCR system (Applied Biosystems, Foster City, CA). PCR-based amplifications were performed using the following primers: NRP1 were 5’-CCGCACCCACGCGA-3’ (forward) and 5’GAAGGAGGGGAACGAGCAATG-3’ (reverse); NSE were 5’-CTGGCTAAATACAACCAGCTCA-3’ (forward) and 5’-CACAGCACACTGGGATTACG-3’ (reverse); KLK3 were 5’-GAGCACCCCTATCAACCCCCTATT-3’ (forward) and 5’-AGCAACCCTGGACCTCACACCTAA-3’ (reverse). Cyclo A and GAPDH were used to normalize RNA input. All primers were purchased from Eurofins Genomics.

**Luciferase Reporter Assay**

NRP1 promoter assay was performed with pLightSwitch Prom vector containing the NRP1 promoter region and Renilla luciferase (#S708087, Active Motif). Briefly, LNCaP cells were plated onto 96-well plates and transfected the next day using Lipofectamine 2000 (Invitrogen) according to manufacturer’s recommendations. LNCaP cells were treated as indicated for 96 h in triplicate and luciferase activity was measured using LightSwitch Luciferase Assay Reagents (Active Motif). Relative light units were calculated as the ratio of Renilla luciferase activity to empty vector.

**Cell Viability and apoptosis assays**

Stably LNCaP, C4-2, 22Rv1 cells overexpressing NRP1 or control vector cells were seeded in 96-well plate at 50% confluence. LNCaP-NE were plated in poly-L-Lysine coated 96-well plate and siRNA transfected the next day using lipofectamine RNaiMax (Invitrogen). The next day, cells were treated with Enzastaurin, docetaxel or combination of the two molecules. Cell viability was determined by using the 3-(4,5-Dimethylthiazol-2-yl)-2,5-diphenyltetrazolium bromide (MTT) assay (Sigma, Saint Quentin Fallavier, France). GI50 values were calculated using the standard curve method (GraphPad Software).

Apoptosis assays were performed using the annexin V-FITC apoptosis detection kit (BD Bioscience) according to the manufacturer’s instruction and analyzed by FACS using CyAn ADP LX7 (Beckman Coulter). Cells that were positive for annexin V but negative for PI were considered to be undergoing apoptosis.

**Immunofluorescence Microscopy**

For dual immunofluorescence, PC3 cells were plated on glass slides overnight. Cells were fixed the next day for 10 min with 4% paraformaldehyde, washed in PBS, then saturated for 30 min with 3% (w/v) BSA. Cells were incubated with anti-human NRP-1 diluted in PBS/ BSA 1% at 4°C overnight. Cells were washed in PBS and incubated at room temperature for 1 h with anti-human PKCα or PKCδ antibodies diluted in PBS/BSA 1%. After several washing, cells were incubated with Alexa Fluor-conjugated 488 or 555 secondary antibodies (Invitrogen) diluted in PBS/BSA 1% for 1h at RT in dark room. Nuclei were labeled with 1 μg/mL DAPI. Cells were mounted with Vectashield mounting medium (Vector Laboratories) and inspected by confocal microscopy. Confocal fluorescence images were acquired using an IX81 inverted Olympus microscope equipped with a DSU spinning disk confocal system (Olympus, France), coupled to an Orca R2 CCD camera (Hamamatsu Corporation, Japan).

Cells were analyzed by acquiring axial z stacks of confocal images (8µm from the base to the top in 0.5µm steps). Residual blurring was removed by spatial deconvolution. Image processing was done using ImageJ software.
