# Supplementary material for: The Neuropilin-1/PKC axis promotes neuroendocrine differentiation and drug resistance of prostate cancer
Source: Br J Cancer. 2022 Dec 22;128(5):918–27. doi: 10.1038/s41416-022-02114-9 (PMC9977768; doi:10.1038/s41416-022-02114-9)
Supplement: Supplementary file 4 — Supplementary Figure 1 [file 41416_2022_2114_MOESM4_ESM.pdf]

Fig. S1

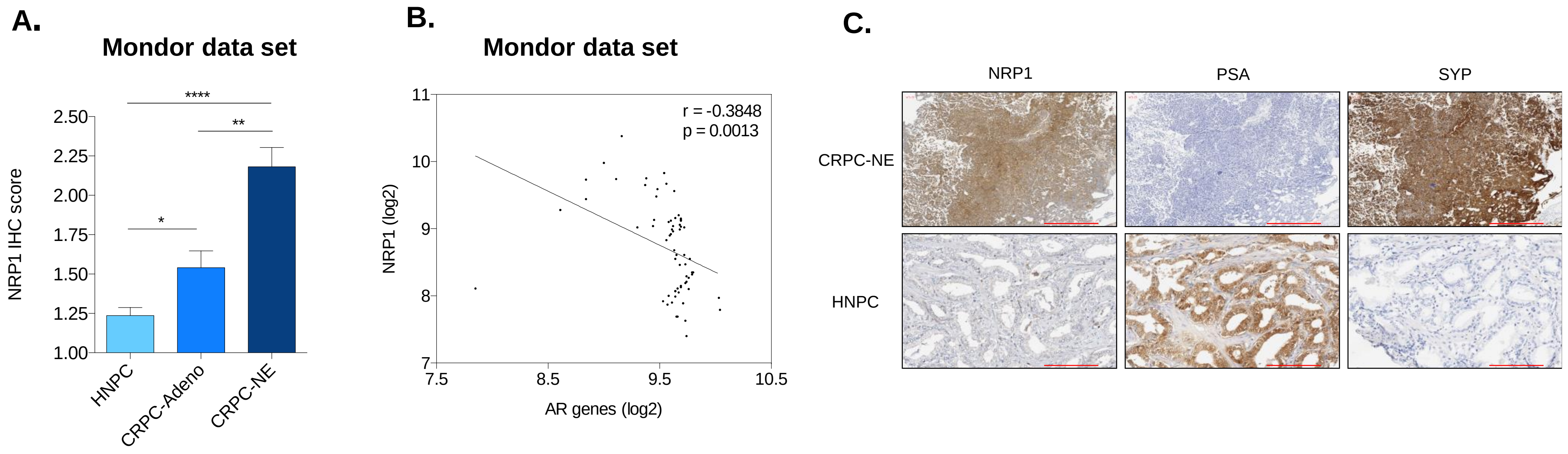

Supplementary Figure 1: **NRP1 expression is associated with PCa progression, inversely related to AR signature and associated with a NED differentiation in human prostate carcinoma.**

A. IHC score for NRP1 protein expression in 169 HNPC, 27 CRPC-Adeno and 21 CRPC-NE. Statistical analyses used a two-tailed $\alpha = 0.05$ level of significance, **, $P < 0.01$; ****, $P < 0.0001$. **B.** Scatter plot shows correlation between *NRP1* mRNA expression and AR signature from Mondor HNPC and CRPC cohort. **C.** Representative IHC for NRP1, PSA and SYP in a HNPC vs CRPC-NE tumor.
