# Supplementary material for: The Neuropilin-1/PKC axis promotes neuroendocrine differentiation and drug resistance of prostate cancer
Source: Br J Cancer. 2022 Dec 22;128(5):918–27. doi: 10.1038/s41416-022-02114-9 (PMC9977768; doi:10.1038/s41416-022-02114-9)
Supplement: Supplementary file 5 — Supplementary Figure 2 [file 41416_2022_2114_MOESM5_ESM.pdf]

Fig. S2

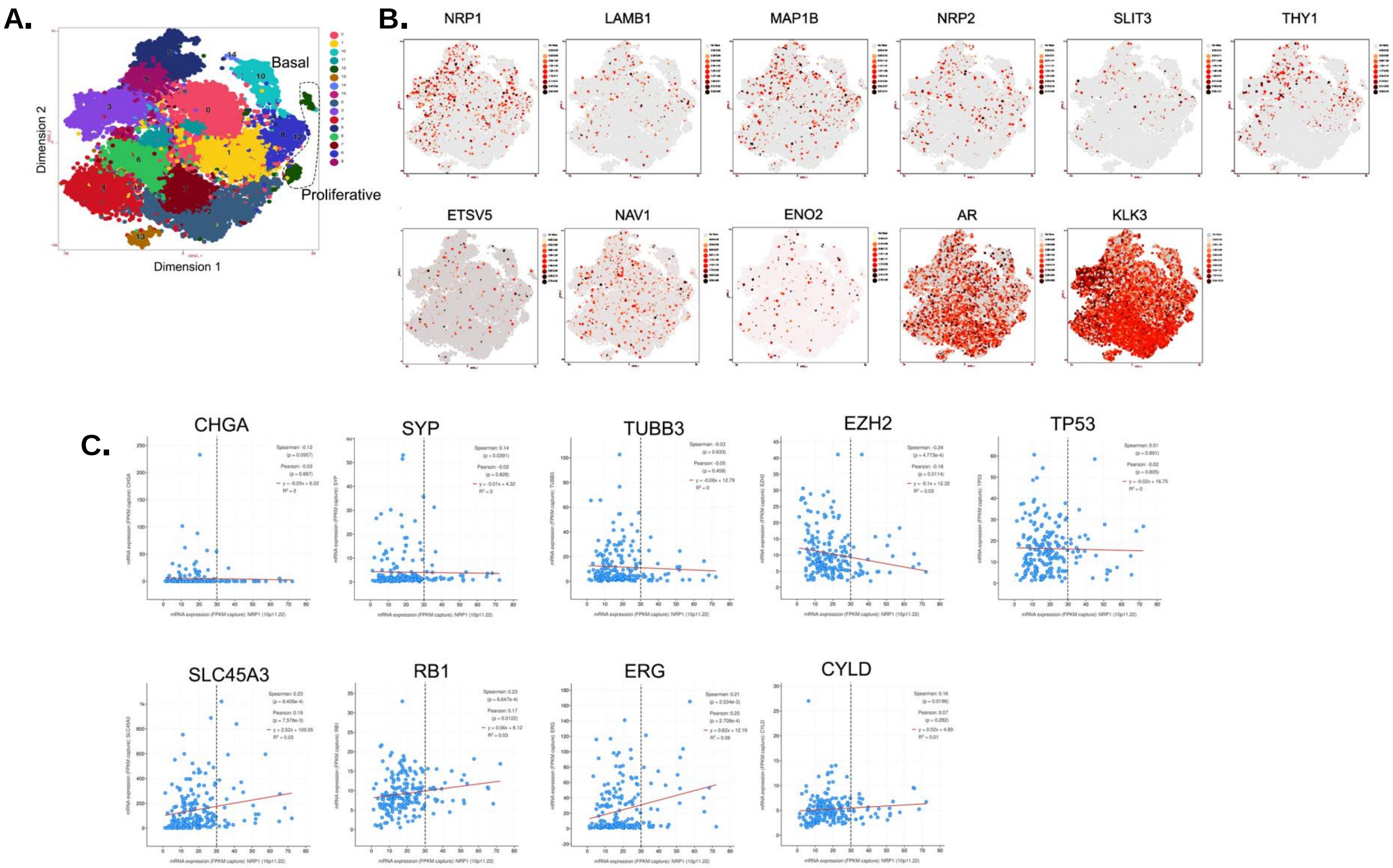

Supplementary Figure 2. scRNAseq analysis of 13 primary tumors confirms NE phenotype in most luminal clusters.
A. TSNE plot shows clustering of malignant cells from integration analysis of 13 tumor biopsy samples across 12 patients **(25). B.** FeaturePlots show expression of selected genes across clusters. Data analyzed using Epithelial PradCellAtlas tool (www.pradcellatlas.com). **C.** Dotplots comparing co-expression of NRP1 (x axis) with defined genes (Y axis) in the SU2C-PCF dataset (208 samples, **ref 32**) using cBioportal tools (https://www.cbioportal.org).
