# Supplementary material for: The Neuropilin-1/PKC axis promotes neuroendocrine differentiation and drug resistance of prostate cancer
Source: Br J Cancer. 2022 Dec 22;128(5):918–27. doi: 10.1038/s41416-022-02114-9 (PMC9977768; doi:10.1038/s41416-022-02114-9)
Supplement: Supplementary file 6 — Supplementary Figure 3 [file 41416_2022_2114_MOESM6_ESM.pdf]

Fig. S3

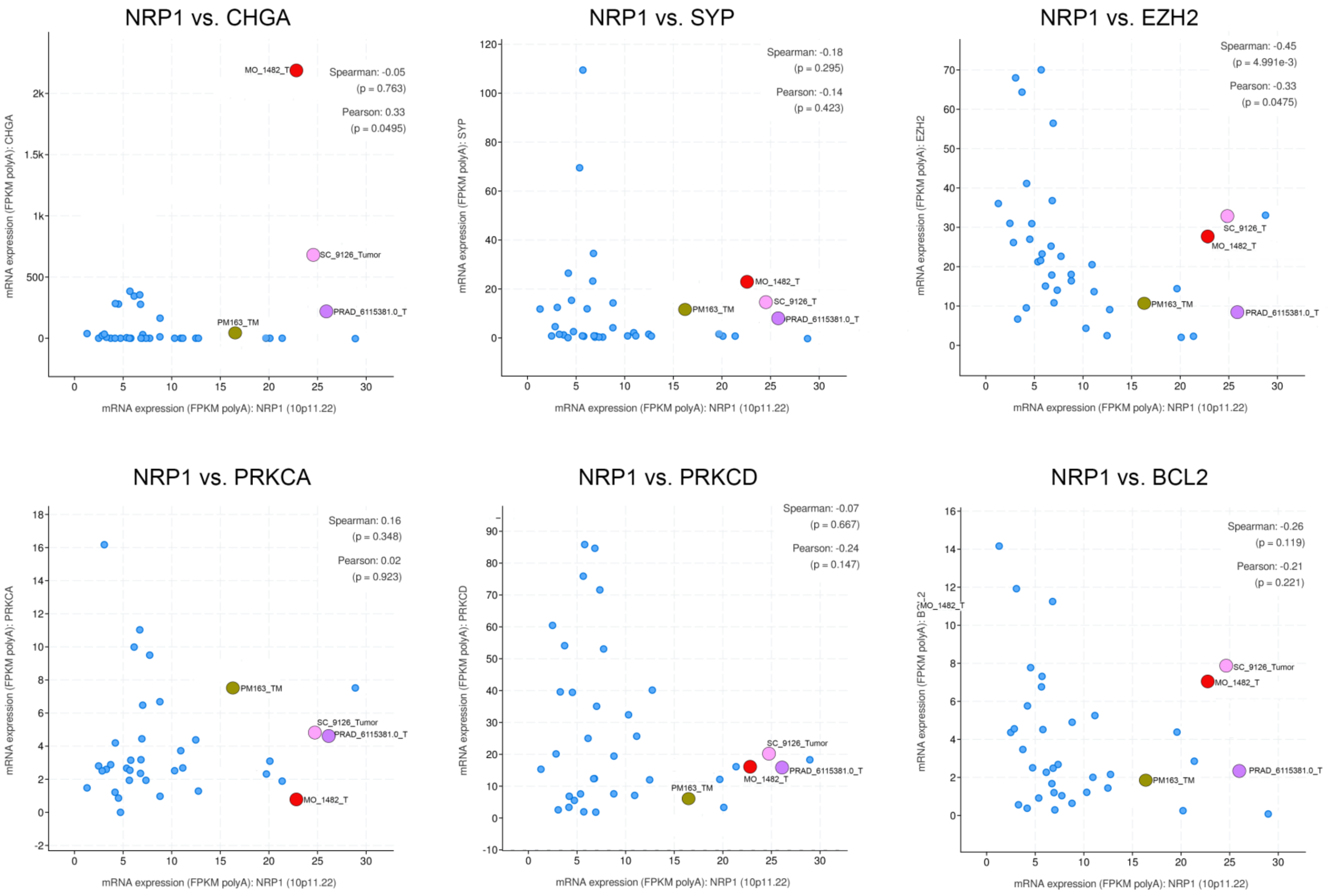

Supplementary Figure 3. Analysis of a High NEPC Score cohort from the SU2C-PCF dataset reveals a subset of NRP1⁺ cells. Dotplots compare co-expression of NRP1 (x axis) with defined genes (Y axis) from a subset of patients **(32)** with high NEPC scores from the SU2C-PCF dataset (39 samples, **ref 32**) using cBioportal tools (https://www.cbioportal.org). Dotted lines in “CHGA” and “SYP” plots mark cutoffs for positivity. Colored dots correspond to the adjacent sample name and are magnified in size to represent putative NRP1+ candidates within the proposed true NE cohort. See **Table S7** for clinical details.
