# Supplementary material for: The Neuropilin-1/PKC axis promotes neuroendocrine differentiation and drug resistance of prostate cancer
Source: Br J Cancer. 2022 Dec 22;128(5):918–27. doi: 10.1038/s41416-022-02114-9 (PMC9977768; doi:10.1038/s41416-022-02114-9)
Supplement: Supplementary file 7 — Supplementary Figure 4 [file 41416_2022_2114_MOESM7_ESM.pdf]

Fig. S4

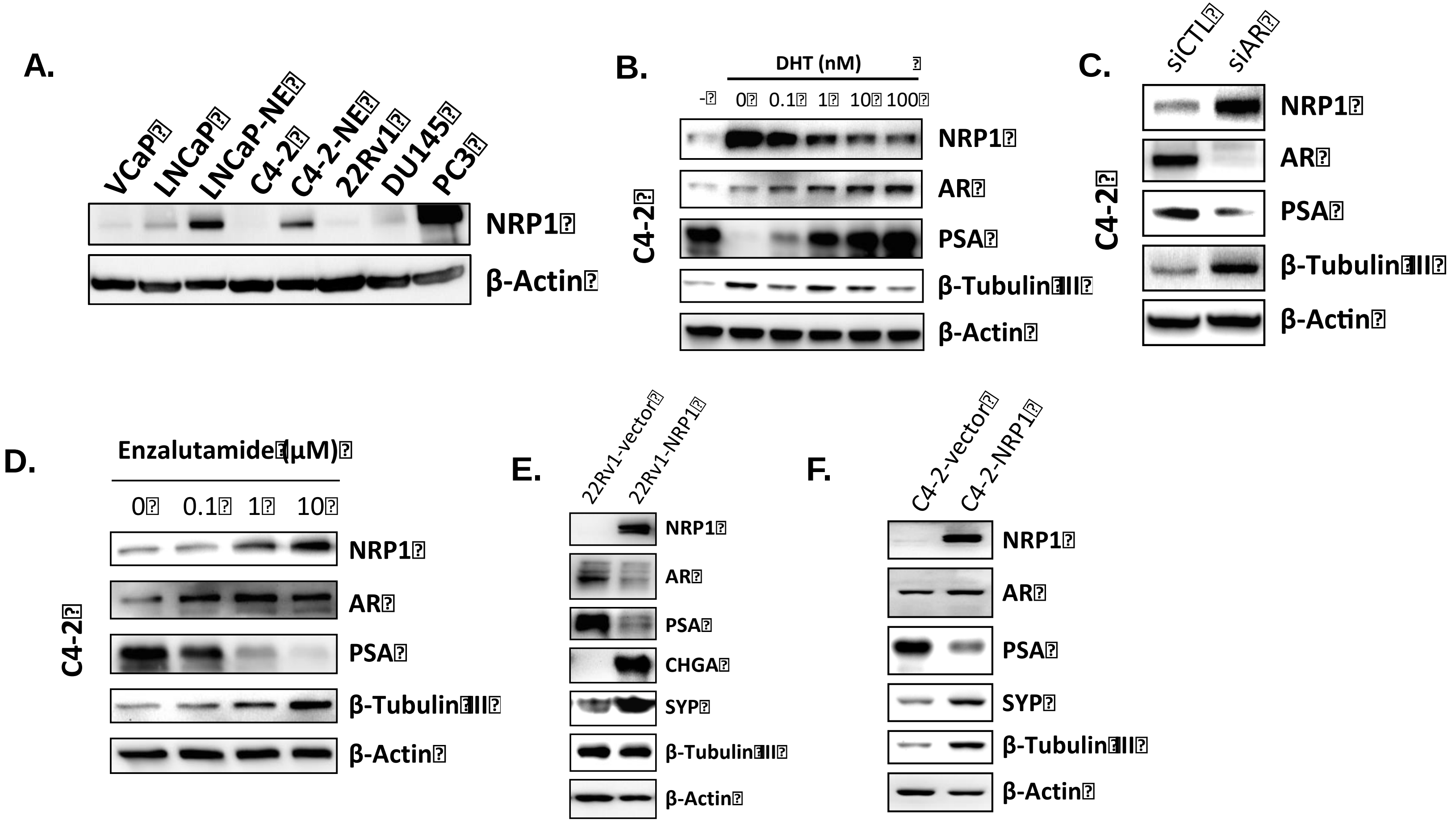

Supplementary Figure 4: **NRP1 promotes NED through regulation of AR axis in a variety of PCa cell lines.**
A. Western blot showing NRP1 protein expression in two PCa androgen-dependent cell lines (VCaP, LNCaP), four androgen-independent cell lines (C4-2, 22Rv1, DU145, PC3), and two neuro-transdifferentiated cell lines upon androgen depletion (LNCaP-NE and C4-2-NE). **B.** Western blot of NRP1, AR, PSA, β-Tubulin III in C4-2 cells treated with DHT at indicated doses for 48h. **C.** Western blot shows NRP1 and other protein expression in C4-2 cells after treatment with AR siRNA or non-targeting siRNA. **D.** Western blot shows NRP1 and other protein expression after enzalutamide treatment in C4-2 cells. **E and F.** Western blots of NRP1, AR, PSA, NE markers CHGA, SYP, β-Tubulin III in stably transfected (**E**) C4-2 and (**F**) 22Rv1 cells overexpressing NRP1 (Right lanes). Cells transfected with empty vector (Left lanes).
