# Supplementary material for: The Neuropilin-1/PKC axis promotes neuroendocrine differentiation and drug resistance of prostate cancer
Source: Br J Cancer. 2022 Dec 22;128(5):918–27. doi: 10.1038/s41416-022-02114-9 (PMC9977768; doi:10.1038/s41416-022-02114-9)
Supplement: Supplementary file 8 — Supplementary Figure 5 [file 41416_2022_2114_MOESM8_ESM.pdf]

Fig. S5

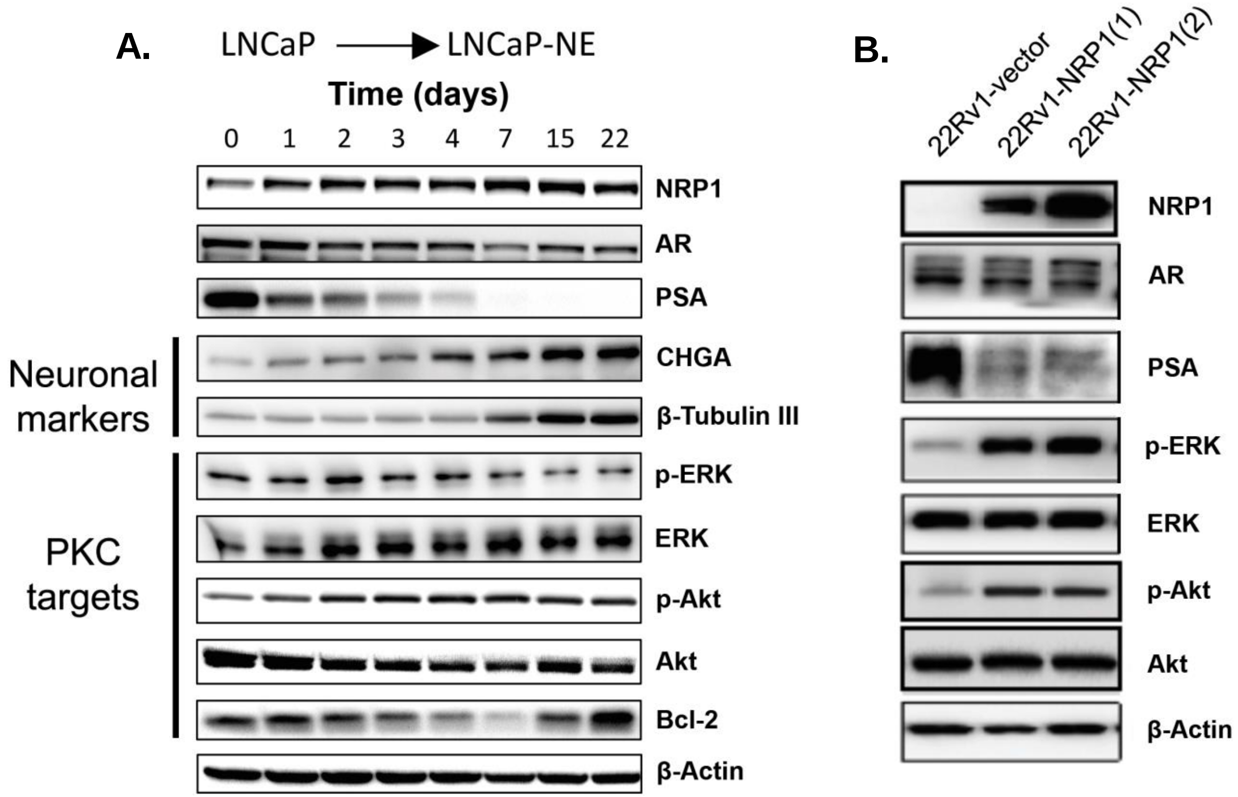

Supplementary Figure 5. Upregulation of PKC downstream targets in LNCaP-NE (A.) and after NRP1 over-expression (B.).
A. Western blot of neuronal markers and PKC-downstream targets in LNCaP cells examined over 22 days following androgen depletion.
B. Western blot of PKC-downstream targets in stably transfected 22RV1 cells overexpressing 2 different NRP1 vectors (22Rv1-NRP1(1 or 2) or with empty vector (22Rv1-vector).
