# Supplementary material for: The Neuropilin-1/PKC axis promotes neuroendocrine differentiation and drug resistance of prostate cancer
Source: Br J Cancer. 2022 Dec 22;128(5):918–27. doi: 10.1038/s41416-022-02114-9 (PMC9977768; doi:10.1038/s41416-022-02114-9)
Supplement: Supplementary file 9 — Supplementary Figure 6 [file 41416_2022_2114_MOESM9_ESM.pdf]

Fig. S6

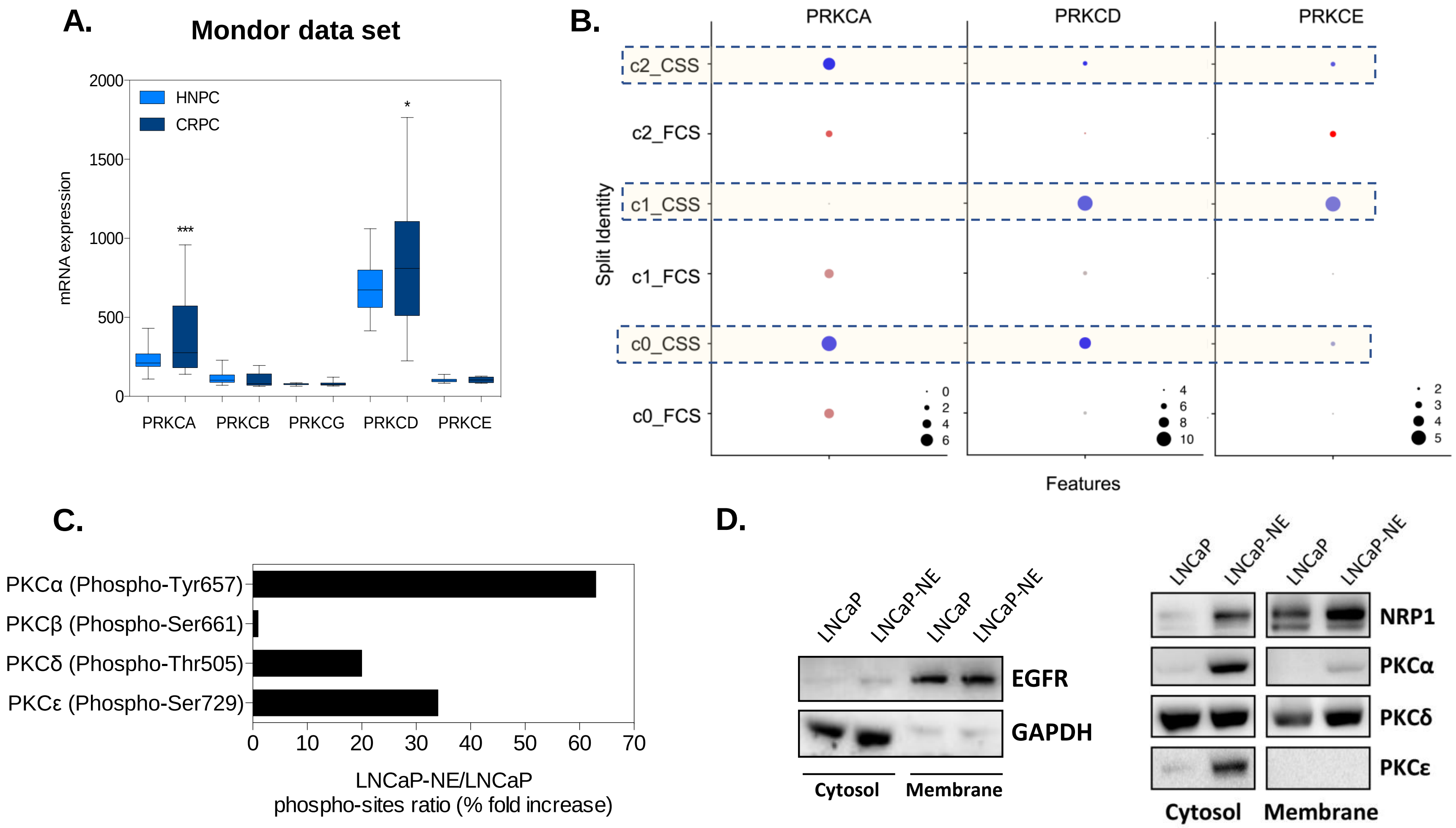

Supplementary Figure 6. Confirmation of transcription, phosphorylation and membrane localization of PKC isotypes in the NE phenotype. **A.** Transcriptomic array results of mRNA expression levels of PKC isoforms in Mondor dataset. **B.** DotPlots show relative expression of defined genes in scRNAseq analysis from GSE205765 (See Materials and Methods for details). FCS (red) and CSS (blue) represent control and hormone-resistant groups, respectively. Scale represents percent expression. Boxed regions highlight CSS clusters. **C.** Bar graph shows antibody microarray levels of phospho-PKC pathway isoforms in LNCaP-NE compared to LNCaP. **D.** Western blots of NRP1, PKCα, PKCδ, PKCε from subcellular cytosol (Left panels) or membrane (Right panels) fractions of LNCaP or LNCaP-NE cells.
