# Supplementary material for: The Neuropilin-1/PKC axis promotes neuroendocrine differentiation and drug resistance of prostate cancer
Source: Br J Cancer. 2022 Dec 22;128(5):918–27. doi: 10.1038/s41416-022-02114-9 (PMC9977768; doi:10.1038/s41416-022-02114-9)
Supplement: Supplementary file 10 — Supplementary Figure 7 [file 41416_2022_2114_MOESM10_ESM.pdf]

Fig. S7

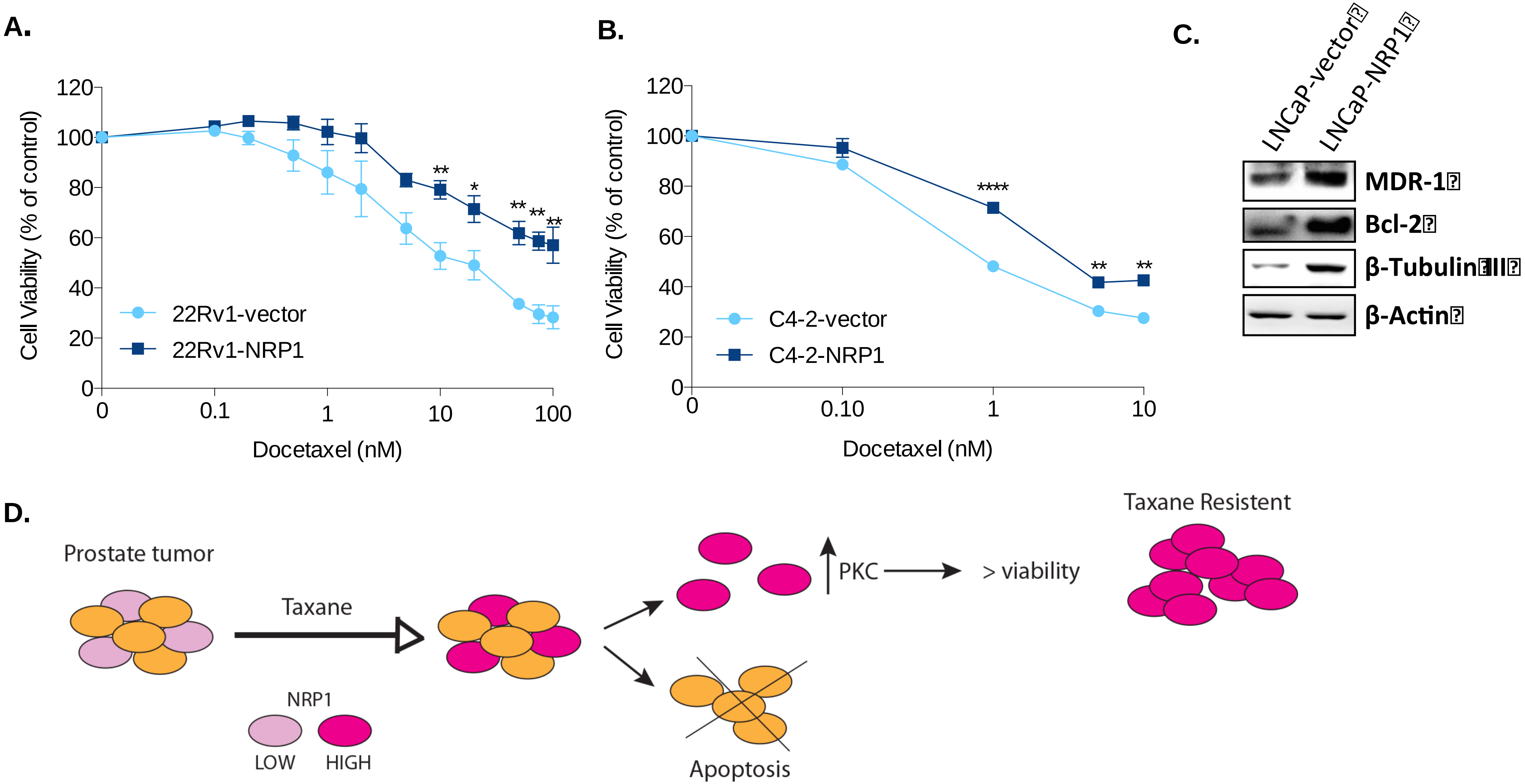

Supplementary Figure 7. NRP1 overexpression confers docetaxel resistance to 22Rv1 and C4-2 cells in vitro.
A and B. Dose-response curves show cell viability in 22Rv1 (**A**) or C4-2 (**B**) clones stably overexpressing NRP1 (squares) or control vector (circles) after 72h incubation with docetaxel (indicated doses on X axis). **C.** Western blot shows MDR-1, Bcl-2 and β-Tubulin III expression in LNCaP cells stably transfected with NRP1 (LNCaP-NRP1) or empty vector (LNCaP-vector). **D.** Model. In this schematic, a subset of pre-treatment tumor cells undergoes increased NRP1 expression upon ADT. NRP1 upregulation drives PKC activation for downstream survival (and drug resistance) in NE tumors. It remains unknown if NRP1 is expressed by a subset or all pre-treatment cells. Further, it remains unknown whether the subset of $NRP1^{+}$ NE tumor cells observed in **Fig S3** reflects a time point in drug resistance or one of several mechanisms used for drug resistance..
